# Retraction: The mechanism of hsa-miR-424-5 combining PD-1 through mTORC signaling pathway to stimulate immune effect and participate in type 1 diabetes

**DOI:** 10.1042/BSR-2019-3800_RET

**Published:** 2022-11-21

**Keywords:** hsa-miR-424-5p, immune effect, mechanism, mTORC signaling pathway, signal molecule, type 1 diabetes

This article is being retracted from *Bioscience Reports* at the request of the Editorial Board following receipt of a notification from a reader, alerting them to the western blots in Figure 5A, which has the appearance of a composite image. In addition, the authors have identified a duplicated flow chart in Figure 3.

The authors had provided replacement images for Figure 5A, however as the original data could not be validated, a Correction could not procced. The Editor-in-Chief, Editorial Board and the authors do not agree to the Retraction.

